# First detection of *Culex tritaeniorhynchus* in Western Australia using molecular diagnostics and morphological identification

**DOI:** 10.1186/s13071-024-06566-1

**Published:** 2024-12-04

**Authors:** Kimberly L. Evasco, Craig Brockway, Tamara Falkingham, Margaret Hall, Nerida G. Wilson, Abbey Potter

**Affiliations:** 1 Environmental Health Directorate, Western Australia Department of Health, 37 Kensington Street, East Perth, Perth, Western Australia 6004 Australia; 2 School of Biological Sciences, University of Western Australia, 35 Stirling Hwy, Crawley, Perth, Western Australia 6009 Australia; 3 Molecular Systematics Unit, WA Museum, 49 Kew St, Welshpool, Perth, Western Australia 6106 Australia

**Keywords:** Encephalitis virus, Surveillance, DNA barcode, Exotic mosquito

## Abstract

**Background:**

*Culex tritaeniorhynchus* has long been considered the primary vector of Japanese encephalitis virus (JEV), but until recently, it was considered exotic to Australia. When the species was detected in the country’s Northern Territory (NT) for the first time, the Western Australia (WA) Department of Health was cognisant of the risk it posed to the State because of the shared border and continuous mosquito habitat adjoining the two jurisdictions. The aim of this study was to undertake intensive mosquito surveillance in the Kimberley region to ascertain whether *Cx. tritaeniorhynchus* was present in WA, define the extent of its distribution and undertake phylogenetic analysis of select specimens to support hypothesized routes of entry into the state.

**Methods:**

Carbon dioxide (CO_2_)-baited encephalitis virus surveillance (EVS) mosquito traps were deployed at various sites throughout the Kimberley region by surveillance officers within the Medical Entomology unit of the Western Australia (WA) Department of Health. Mosquitoes were then morphologically identified, and a subset of four specimens were confirmed as *Cx. tritaeniorhynchus* by molecular identification using Cytochrome Oxidase I (COI) DNA data and phylogenetic analysis.

**Results:**

From 31 March 2021 to 30 May 2024, a total of 211 female *Cx. tritaeniorhynchus* specimens were collected from 21 unique trap sites in the Kimberley’s Shire of Wyndham-East Kimberley (SWEK). Four COI DNA barcode regions were amplified and successfully sequenced for analysis. These sequences fell within a clade recognised as *Cx. tritaeniorhynchus* and specifically all sequences were in a clade with other specimens from the NT and Timor-Leste.

**Conclusions:**

This study represents the first detection of *Cx. tritaeniorhynchus* in WA. Given the widespread nature of trap sites that yielded the species and consecutive seasons over which it was observed, the authors surmise that *Cx. tritaeniorhynchus* is now established within the northeast Kimberley region. The findings are significant given the detection of the species coincides with the first significant outbreak of JEV activity on mainland Australia involving an estimated 45 human cases of Japanese encephalitis, 80 impacted commercial piggeries and widespread feral pig activity. Although the role that *Cx. tritaeniorhynchus* may play in JEV transmission into the future is not yet understood, it presents a potential risk to public health in the region.

**Graphical abstract:**

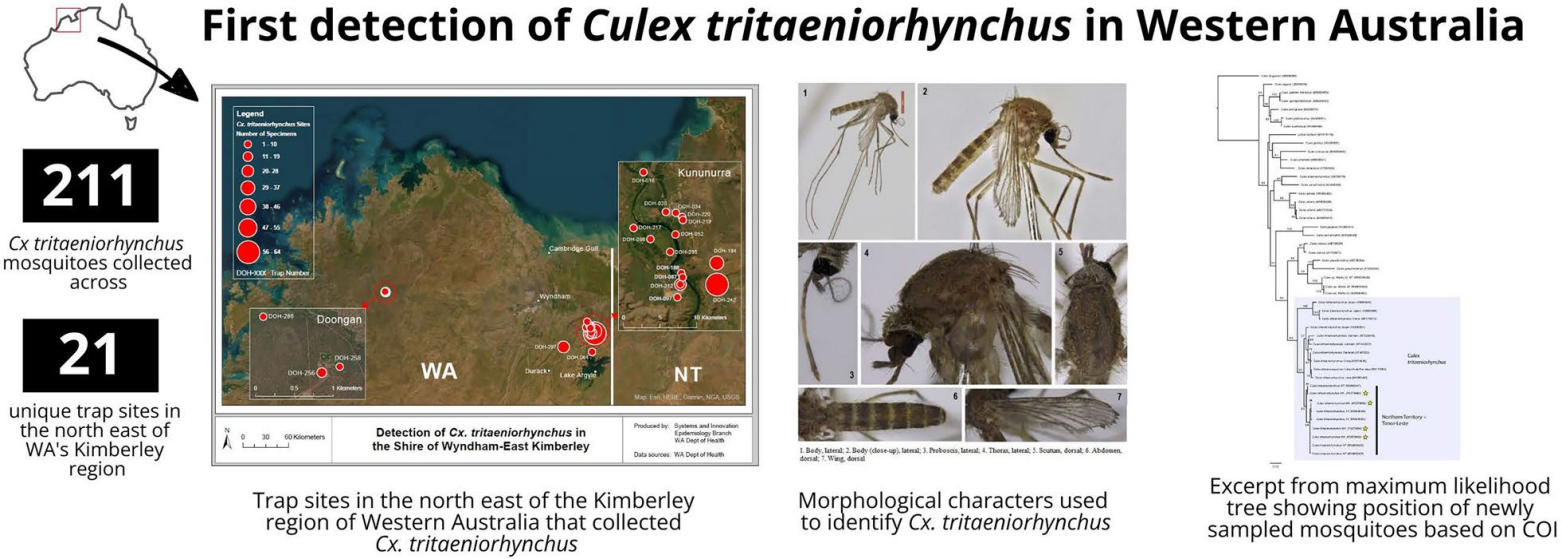

**Supplementary Information:**

The online version contains supplementary material available at 10.1186/s13071-024-06566-1.

## Background

*Culex tritaeniorhynchus* has long been considered the primary vector of Japanese encephalitis virus (JEV) [11, 19, 22]. Although geographic distribution of the vector has traditionally been associated with Southeast Asia, *Cx. tritaeniorhynchus* has now been detected in at least 41 countries, across 5 different continents [47]. Most recently, Lessard et al. [30] described the first records of *Cx. tritaeniorhynchus* being detected in Australia. Specimens were collected in February and March 2020 from the Northern Territory (NT) before being confirmed by morphological identification and DNA barcoding [30].

Recent detections of *Cx. tritaeniorhynchus* in the NT are particularly significant because of the unexpected emergence and spread of JEV in Australia. On March 4, 2022, Japanese encephalitis (JE) was declared a ‘Communicable Disease Incident of National Significance’ (CDINS) [7]. Over the course of the outbreak, a total of 45 confirmed and probable human cases of JE were notified, 7 of which were fatal [15]. Japanese encephalitis virus activity was detected in over 80 commercial piggeries across Queensland (QLD), New South Wales (NSW), Victoria and South Australia (SA) [3]. Serological evidence of prior infection with JEV and/or detection of the virus by polymerase chain reaction (PCR) on tonsil specimens was also found in feral pigs from the NT, SA, northern QLD and the Kimberley region of Western Australia (WA) [3, 15]. This outbreak represents the first evidence of JEV on mainland Australia since sporadic activity was previously recorded on the northern tip of Cape York Peninsula in 1998 and 2004 and a fatal case of JE on the Tiwi Islands, NT, in 2021 [23, 51, 54]. The latter is now hypothesized to be the sentinel case for the most recent outbreak on mainland Australia [54].

When *Cx. tritaeniorhynchus* was first detected in the NT, the WA Department of Health’s (the Department) Medical Entomology unit was cognisant of the possibility that distribution may expand over time into WA, given the shared jurisdictional border. This risk was heightened by the knowledge that a homogeneous ecosystem exists between the NT and the neighbouring Kimberley region, conducive to supporting *Cx. tritaeniorhynchus* breeding and harbourage [14]. The aim of this study was to ascertain whether the species was present in WA, define the extent of its distribution and undertake phylogenetic analysis of select specimens to support hypothesized routes of entry into the state.

This publication documents the first confirmed detections of *Cx. tritaeniorhynchus* in WA at a time and location when serological evidence of prior exposure to JEV in sentinel chickens and feral pigs was demonstrated, indicating the virus had been circulating within the environment (unpublished data, WA Department of Health, 2023). Although the role that *Cx. tritaeniorhynchus* may have played in JEV transmission in the NT and WA during the recent Australian outbreak is not yet understood, it is anticipated that the establishment of this highly competent JEV vector in an environment conducive to endemic flavivirus activity will have potentially significant public health implications into the future.

## Methods

### Description of study sites

**Fig. 1:**
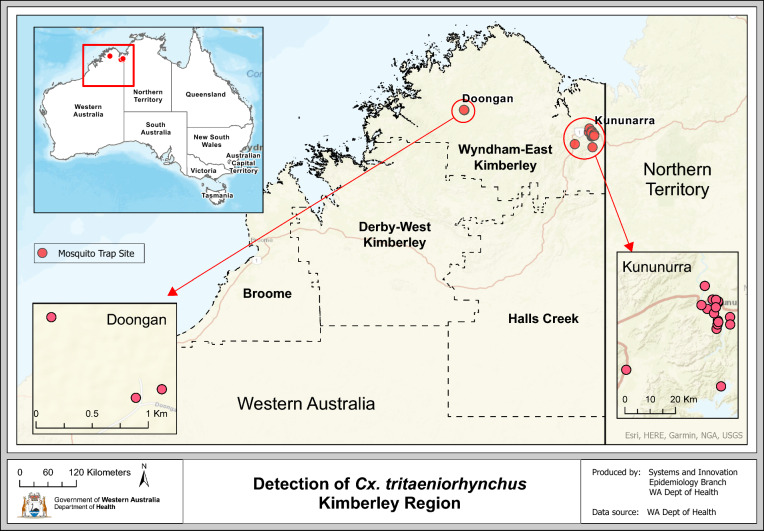
Map of adult mosquito trap site locations in the Kimberley region of Western Australia, showing where *Culex tritaeniorhynchus* was detected

Western Australia (WA) is the largest state in Australia, occupying the whole western third of the country (2.53 million km^2^) [18]. Despite its substantial size, WA’s population remains at 2.95 million, with 75% of individuals residing in the capital city of Perth, in the state’s southwest [6]. Much of WA remains remote and challenging to access. The Kimberley region (Fig. 1), located in WA’s far north, consists of four local government authorities (LGAs), including the Shires of Broome, Derby-West Kimberley, Halls Creek and Wyndham-East Kimberley (SWEK). The focus of this study will be on SWEK in WA’s northeast, with a population of 7477 [5]. The eastern aspect of SWEK borders onto the NT. Unlike Perth’s Temperate climate, SWEK experiences a tropical climate characterised by a distinct wet (November–April) and dry season (May–October) [4]. Mosquito trap site locations within SWEK, where *Cx. tritaeniorhynchus* was detected, can be observed in Fig. 1 and in more detail in Fig. 4. Traps were also set throughout the remaining three LGAs in the Kimberley (Supplementary Fig. 1), but as *Cx. tritaeniorhynchus* was not detected, and the number of trap sites were numerous, they will not be discussed in detail here.

### Mosquito collection

Carbon dioxide (CO_2_)-baited encephalitis virus surveillance (EVS) traps were deployed at trap sites on a range of dates from 1 March 2021 to 30 May 2024 by surveillance officers within the Department’s Medical Entomology unit. Each CO_2_-baited EVS trap contained ~ 1 kg of dry ice pellets as well as a light to attract mosquitoes. Mosquitoes were forced into the trap net and catch bucket by a downward blowing fan, powered by two rechargeable ‘D’ cell batteries. Traps were set as close to sunset (5:00 p.m.–6:00 p.m. GMT) as possible and collected again the following morning around sunrise (4:45 a.m.–6:00 a.m. GMT). Upon collection, trap buckets were immediately placed into a portable insulated container with dry ice to ‘knock down’ or kill any mosquitoes. The date set, trap site name, geocode and distance from the nearest water source margin where *Cx.* *tritaeniorhynchus* was predicted to be breeding was recorded for all specimens. Water source margins were considered the closest suitable habitat that supported *Cx.* *tritaeniorhynchus* oviposition. This varied by trap location and included permanent or ephemeral wetlands that held water over the northern wet season, characterised by shallow depths and emergent vegetation. In other cases, it represented anthropogenic irrigation channels, drainage ditches and flood irrigation land. All trap specimens were then transferred into separate sample collection containers (Sarstedt flat-base, translucent, screw top) and stored in a portable − 80 °C freezer in Kununurra before transport to Perth could be arranged. On the day of transport, sample collection containers were transferred to a portable Styrofoam cooler box filled with dry ice. A Medical Entomology officer then accompanied the samples by air, as hand carry, from Kununurra to Perth, where they were then placed into a commercial − 80 °C freezer at the Medical Entomology laboratory until processing was undertaken. Cold chain transport was maintained throughout this time as part of the standard transport and storage procedure for preservation of the integrity of specimens.

### Morphological identification

After preliminary morphological identification of 12 *Culex* mosquitoes (trapped over the nights of 28 and 29 March 2022) using a standard dissecting stereo microscope failed to key out to any previously recorded species endemic to northern WA, a leg was removed from each of four randomly chosen specimens for DNA extraction [31]. The mosquito specimens were then pinned for accurate morphological identification. Mosquitoes were examined under a Leica M80 dissecting microscope (Leica Microsystems Pty Ltd.) and identified using Lee’s Culicidae of the Australasian Region [29]. Identification was further confirmed using morphological identifiers referenced by Lessard et al. [30]. Photographs of mosquitoes were taken with a Leica M205 C stereo microscope (Leica Microsystems Pty Ltd) and stacked in Leica Application Suite V4.12.0 to obtain high-resolution, clear images. Once both morphological and molecular identification had been confirmed on samples collected in March 2022, historical unidentified *Culex* samples (collected 31 March and 1 April 2021) were re-examined and unpinned under a standard dissecting microscope and morphologically confirmed to also be *Cx. tritaeniorhynchus*. All other mosquitoes collected after 28 and 29 March 2022 were morphologically identified using the above methods, ensuring that all identifying characters were present.

### DNA extraction, amplification and sequencing

DNA was extracted from leg tissue from four specimens using a DNeasy Blood and tissue kit (Qiagen) following the manufacturer’s instructions. A 658-bp partial mitochondrial cytochrome oxidase subunit I (COI) gene was amplified by PCR in the Molecular Systematics Unit (Western Australian Museum) and sequenced at the Australian Genomic Research Facility (AGRF) Perth node, using the COI primers LepF (5′-ATTCAACCAATCATAAAGATATTGG-3′) and LepR (5′-TAAACTTCTGGATGTCCAAAAAATCA-3′) [21].

### Sequence analysis

Bidirectional sequences were assembled and edited in Geneious Prime 2022.1.1. Extraneous primer sequences were removed prior to analysis. The DNA sequences were queried against publicly accessible sequences in GenBank using the Basic Local Alignment Search Tool (BLAST) for veracity and subsequently analysed in a phylogenetic framework with an appropriate reference mosquito data set [30]. Sequences were analysed under a maximum likelihood criterion in IQ-tree web server [49], with the best model fit calculated as GTR + F + I + G4, with nodes tested with 1000 ultrafast bootstraps [35]. Genetic distances were calculated via tree-based estimates of identical bases in Geneious Prime 2022.1.1.

## Results

### Morphological analyses

**Fig. 2:**
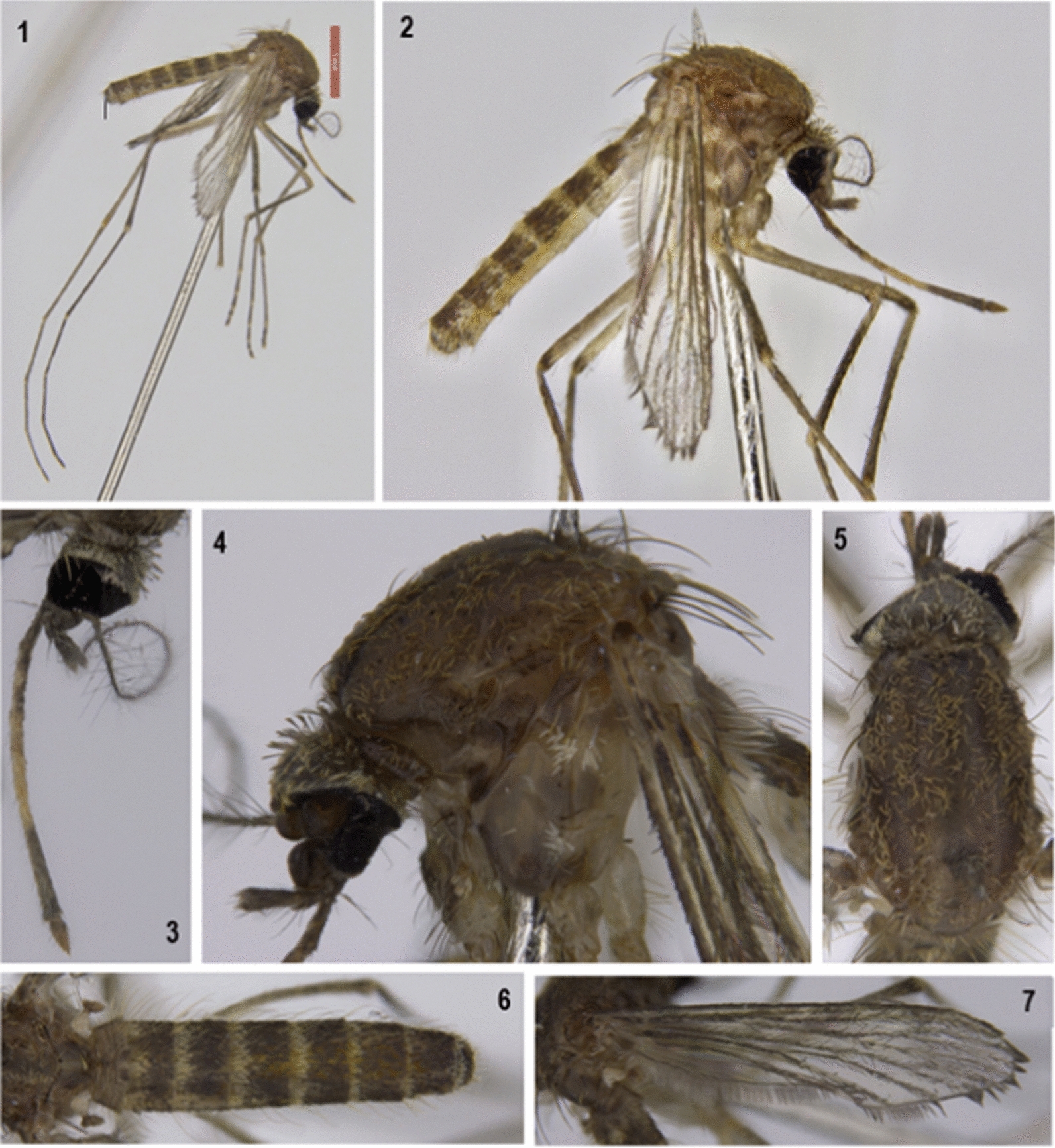
Morphological characters used to identify *Culex tritaeniorhynchus* adult mosquito specimens

Morphological identification of *Cx. tritaeniorhynchus* was undertaken using diagnostic keys by Lee et al. [29] and Lessard et al. [30]. These resources informed differentiation between other morphologically similar species previously recorded in the region: *Culex crinicauda, Culex* Marks sp. No. 92 and the most similar *Culex* Marks sp. No. 32 [30]. The distinguishing morphological characters are shown in Fig. 2 and detailed below:

Head: Vertex with erect forked scales predominantly brown (Fig. 2.1). Proboscis with a broad, median pale band (Fig. 2.3). Scutum with unicolorous brown scaling, acrostichal setae present, no patches of white scales (Fig. 2.5).

Thorax: Distinctive reduced scale patches on pleura (Fig. 2.4), postspiracular scales absent, mesokatepisternal scales and forecoxa scales present.

Abdomen: Tergites brown with complete pale basal bands (Fig. 2.6), sternites pale (Fig. 2.2).

Legs: Almost entire length of posterior hind femora pale, dark at apical end, anterior surface of mid tibia brown with no longitudinal pale line (Fig. 2.2).

Wings: Dark scaled with row of pale scales posterior to the leading edge of the base of costa (Fig. 2.7).

### Molecular analyses

**Fig. 3:**
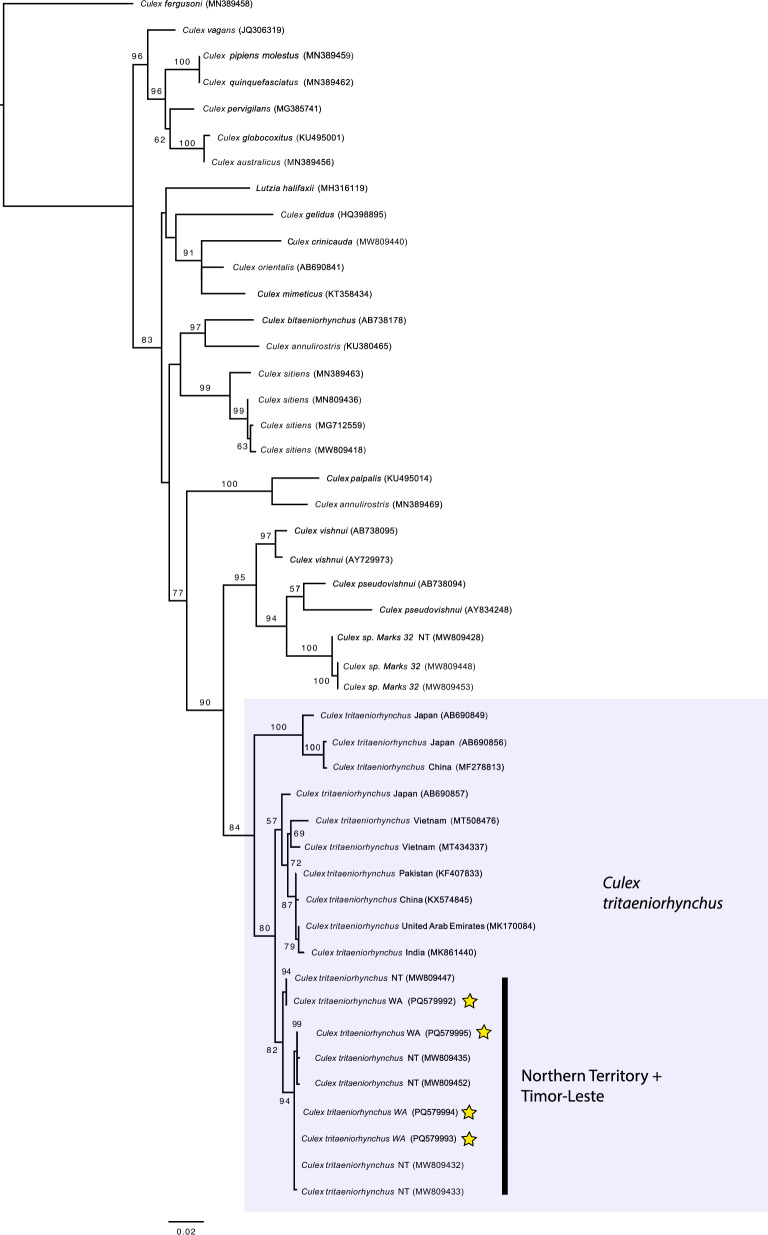
A maximum likelihood tree showing the position of the newly sampled mosquitoes based on COI data. Stars represent samples from this study. Shaded box represents *Culex tritaeniorhynchus*. Bootstrap values < 50 have been removed as well as some interior node values for better visualization

Following initial morphological examination, confirmation of identification was sought through molecular analysis. Four mosquitoes were randomly selected from the 12 initial specimens that had been collected and identified as *Cx. tritaeniorhynchus* and were successfully sequenced for partial mitochondrial COI (the barcoding region) at the Molecular Systematics Unit, WA Museum. The four query sequences were all nested within a monophyletic clade recognised as *Cx. tritaeniorhynchus* (Fig. 3). Three different haplotypes were represented in the newly generated data, and the divergence of the new samples ranged from 0 to 1%, representing a high degree of sequence conservation. All target sequences were in a monophyletic clade with other specimens from the NT and Timor-Leste (Fig. 3). Three target sequences were identical to two from the NT (MW809447, MW809432) and one sequence was equally close (0.2% divergent) to sequences from Timor-Leste or the NT.

### Trap location and timing

From 1 March 2021 to 30 May 2024, a total of 444 unique trap sites (> 1568 trapping events) were surveyed for adult mosquitoes across the four LGAs in the Kimberley region (Table 1; Additional file 1: Figure S1).

**Table 1:** Total number of unique traps and ^a^trapping events undertaken in each local government authority in the Kimberley region from 1 March 2021 and 30 May 2024

	Shire				Total
	Wyndham-East Kimberley	Halls Creek	Derby-West Kimberley	Broome	
No. unique traps	211	84	84	65	444
No. trapping events	970	223	151	112	1568

^a^A trapping event represents a single trap set on a given night

Significantly, a total of 211 *Cx. tritaeniorhynchus* specimens were collected across 21 unique sites in SWEK over 24 trapping events (Table 2). *Culex tritaeniorhynchus* was not detected in the Shires of Broome, Derby-West Kimberley or Halls Creek. Table 2 details the date each of the 24 traps were set, the unique trap code, latitude/longitude, number of *Cx. tritaeniorhynchus* collected and the distance the trap was located from water source margins where the mosquitoes were predicted to be breeding. All specimens were female. All *Cx. tritaeniorhynchus* mosquitoes were collected between the months of January and April, across the 38-month period, with the exception of those from Doongan, which were collected in May. The number of trapping events that detected *Cx. tritaeniorhynchus* increased over consecutive years, from 3 trapping events in 2021 and 2022, respectively, to 6 in 2023, before peaking at 12 in 2024. *Culex tritaeniorhynchus* was detected at site codes DOH-212, DOH-217 and DOH-020 over two consecutive mosquito seasons.

**Table 2:** *Culex tritaeniorhynchus* surveillance data, including trap date, location, number of specimens and distance from water source margins

Trap date	Trap number	Latitude	Longitude	Number specimens	Sex	Distance from water source margins (m)
31-Mar-21	DOH-187	15°51′ 33′′ S	128°45′ 00′′ E	3	F	20.0
31-Mar-21	DOH-188	15°51′ 14.0′′ S	128°44′ 54.0′′ E	2	F	99.0
1-Apr-21	DOH-097	15°53′ 01.2′′ S	128°44′ 36.7′′ E	3	F	10.0
28-Mar-22	DOH-212	15°52′ 04.4′′ S	128°44′ 50.1′′ E	9	F	28.0
28-Mar-22	DOH-217	15°47′ 55.7′′ S	128°41′ 23.2′′ E	1	F	9.0
29-Mar-22	DOH-064	16°05′ 18.1′′ S	128°45′ 25.8′′ E	2	F	20.0
1-Feb-23	DOH-098	15°48′ 44′′ S	128°42′ 37′′ E	2	F	51.0
1-Feb-23	DOH-212	15°52′ 04.4′′ S	128°44′ 50.1′′ E	24	F	28.0
1-Feb-23	DOH-217	15°47′ 55.7′′ S	128°41′ 23.2′′ E	9	F	9.0
1-Feb-23	DOH-219	15°47′ 20′′ S	128°45′ 00′′ E	2	F	8.8
1-Feb-23	DOH-220	15°47′ 07′′ S	128°44′ 58′′ E	4	F	5.5
2-Feb-23	DOH-020	15°46′ 44.6′′ S	128°43′ 45.9′′ E	1	F	188.0
12-Jan-24	DOH-187	15°51′ 33′′ S	128°45′ 00′′ E	1	F	20.0
23-Jan-24	DOH-297	16°01′ 46.3′′ S	128°25′ 18.7′′ E	26	F	0
26-Feb-24	DOH-020	15°46′ 44.6′′ S	128°43′ 45.9′′ E	1	F	188.0
26-Feb-24	DOH-295	15°49′ 40′′ S	128°44′ 04′′ E	7	F	22.0
27-Feb-24	DOH-016	15°43′ 49.5′′ S	128°42′ 06.9′′ E	1	F	24.0
29-Feb-24	DOH-034	15°46′ 48.6′′ S	128°44′ 30.0′′ E	5	F	24.2
29-Feb-24	DOH-052	15°48′ 24.2′′ S	128°44′ 28.0′′ E	1	F	38.0
29-Feb-24	DOH-184	15°50′ 29.8′′ S	128°47′ 30.7′′ E	30	F	51.1
29-Feb-24	DOH-242	15°52′ 05.0′′ S	128°47′ 32.1′′ E	64	F	25.0
30-May-24	DOH-256	15°22′ 48′′ S	126°18′ 38′′ E	11	F	525.0
30-May-24	DOH-258	15°22′ 46′′ S	126°18′ 46′′ E	4	F	311.0
30-May-24	DOH-286	15°22′ 25′′ S	126°18′ 14′′ E	1	F	29.2
Total number of *Cx. tritaeniorhynchus* specimens collected				211		

**Fig. 4:**
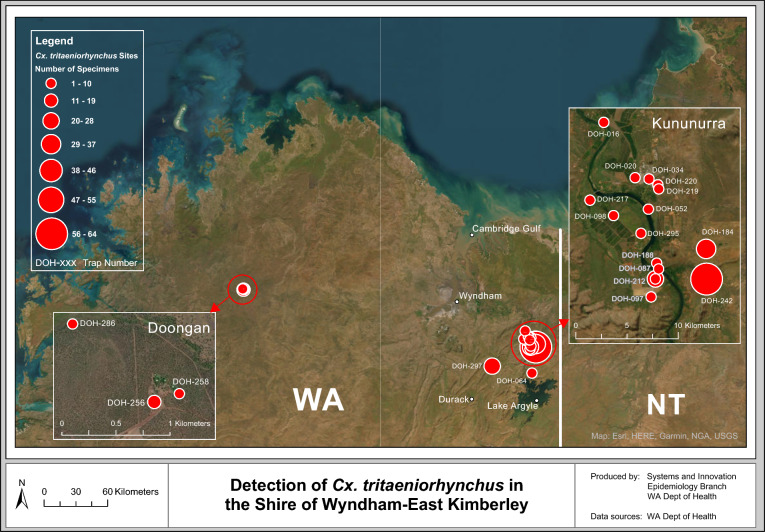
Trap site locations and number of *Culex tritaeniorhynchus* specimens collected in the Shire of Wyndham-East Kimberley, Western Australia (March 2021–May 2024)

The 21 unique trap site locations and number of *Cx. tritaeniorhynchus* specimens collected per trap are shown in Fig. 4. Fifteen of the sites were situated within the Town of Kununurra in the east of SWEK, close to the NT border. Outside of Kununurra, positive trap sites extended south to Dead Horse Springs on the northern aspect of Lake Argyle (DOH-064), southwest towards Durack (DOH-297) and ~ 240 km northwest to Doongan (DOH-256, 258 and 286).

### Trap location climatic conditions

Average climatic conditions (rainfall and temperature) for both the Doongan and Kununurra weather stations are provided in Table 3, as these variables have a significant impact on mosquito breeding and will be further discussed in relation to the results in this study [12]. Of note, Doongan (DOH-256, 258 and 286) experienced unseasonably high rainfall (39 mm) the night trapping took place, exceeding the previous record for highest daily rainfall for May, recorded in 2013 (35.8 mm). In the 2 days leading up to trapping in Doongan, a rapid rise in humidity (47–91%) was also observed [12].

**Table 3:** Temperature and rainfall data for Doongan and Kununurra weather stations

	Jan	Feb	March	Apr	May
Doongan, Western Australia (Station no. 1025)					
Temperature					
Lowest daily temperature (°C)	22.6	30.5	30.2	29.8	28.7
Highest daily temperature (°C)	40.0	39.06	39.0	38.5	36.5
Monthly mean (°C)	33.1	32.9	33.4	33.3	31.3
Rainfall					
Mean (mm)	306.6	237.2	202.2	44.7	13.5
Highest daily (mm)	167.2	132.6	243.0	97.5	35.8
Kununurra, Western Australia (Station no. 2056)					
Temperature					
Lowest daily temperature (°C)	26.4	26.6	24.9	25.3	20.2
Highest daily temperature (°C)	43.1	41.7	43.0	40.4	38.5
Monthly mean (°C)	35.8	35.2	35.8	35.5	32.9
Rainfall					
Mean (mm)	216.8	215.2	143.0	31.6	6.6
Highest daily (mm)	162.2	163.8	165.8	73.6	42.8

Reference: [12].

## Discussion

This is the first published record of *Cx. tritaeniorhynchus* in WA and the second in Australia [30]. Surveillance efforts over the 38-month period yielded 211 specimens, morphologically identified as *Cx.* *tritaeniorhynchus*, four of which were confirmed by molecular analysis. As all target sequences from the mosquitoes analysed in this study were found to be in a clade with other specimens from the NT and Timor-Leste (Fig. 3), it is surmised that the introductory pathway into WA is likely to have occurred via one of these jurisdictions. The results of this study indicate that the highly competent JEV vector has been present in WA since at least March 2021. Based on ongoing detections in SWEK over the surveillance period, and repeated detection at three unique sites over two consecutive mosquito seasons, *Cx.* *tritaeniorhynchus* is now presumed to be established in the region. As no *Cx.* *tritaeniorhynchus* was detected in the Shires of Broome, Derby-West Kimberley or Halls Creek, despite intensive surveillance, distribution is not thought to extend to these other LGAs at this time.

### Potential routes of entry to WA

*Culex tritaeniorhynchus* was first observed in WA on 31 March 2021 at a trap site 22.3 km from the NT border, approximately 12 months after the species was first detected in the NT [30]. Given the timeline alignment and molecular results, we hypothesize that the most likely route of introduction to WA was due to expansion of species distribution from the NT after its initial incursion from Timor-Leste. This is supported by the phylogenetic analyses, which indicated three of the four sequences from WA specimens were identical to those sequences from the NT, and the final one was equally close (0.2% divergent) to sequences from Timor-Leste or the NT. Lessard et al. [30] determined that the *Cx. tritaeniorhynchus* specimens detected in the NT were most similar (99.7% nucleotide identity) to a specimen sequenced from Dili, Timor-Leste [30]. A distance of only 465 km divides Melville Island (Tiwi Islands) near Darwin and Timor-Leste; as such, it is plausible that the initial incursion may have been the result of windblown or transport-mediated movement of the species into the NT before establishment and distribution expanded further inland to Katherine [30]. Interestingly, the molecular diagnostics undertaken by Lessard et al. [30] placed the *Cx. tritaeniorhynchus* specimens from the NT within a larger clade that included Darwin, Timor, Japan and China, whilst the molecular analysis undertaken in this study indicated that the WA specimens were nested in a clade consistent with sequences from Darwin and/or Timor-Leste. Given a continuous ecosystem exists between the NT and the neighbouring Kimberley region, geographic expansion of the species is predicted to have then occurred over time [14, 16]. Spread may have been expedited by the transport of adult mosquitoes or larvae/pupae via aircraft, road transport, cargo ship or boat. Frequent flights are scheduled between Darwin and Kununurra in addition to daily freight services provided by road trains between Darwin, Katherine and Kununurra. Although less plausible, it is also possible that carriage of larvae/pupae may have occurred via cargo ship into Wyndham, 73 km northwest of Kununurra, as it is the only deep-water port between Broome and Darwin. In a study undertaken by Nie et al. [36], *Cx. tritaeniorhynchus* was documented as being one of the most dominant mosquito species to be detected on ships arriving to China from abroad [36]. However, Wyndham did not receive any vessels whose port of origin was recorded as Dili or Darwin during 2021–2022, and only one cruise vessel from Darwin was confirmed to have docked at Wyndham Port during 2022–2023 [3]. Entry via unauthorised vessel from international points of origin may also present a potential route of introduction; however, the authors do not have information on the number of arrivals during the relevant time period. As *Cx tritaeniorhynchus* was not detected in the town of Wyndham, despite numerous trapping events taking place at the location over the study period, this remains a less likely scenario.

Alternatively, *Cx. tritaeniorhynchus* may have entered WA as a direct windblown incursion from overseas. This study demonstrates the presence of *Cx. tritaeniorhynchus* in Doongan in WA’s far northern Kimberley region, which is ~ 600 km from Timor-Leste. Given *Cx. tritaeniorhynchus* has previously been recorded as flying 200–500 km over sea waters in the Northwest Pacific, and wind dispersal of ~ 1000 km between continental Asia and Japan, it is possible that windblown mosquitoes may have made it to the northern-most tip of WA [1, 2]. When challenged in a flight mill simulating atmospheric conditions at 1000 m above sea level, *Cx. tritaeniorhynchus* females were capable of flying continuously for up to 38 h [1]. In Australia, *Culex annulirostris* Skuse is considered a morphological and biological counterpart of *Cx. tritaeniorhynchus* [40]. Dispersal studies in Australia have demonstrated that *Cx. annulirostris* can travel between 594 and 648 km at night by ascending from surface air into the planetary boundary layer [27]. In North America, it has also been suggested that *Culex tarsalis*, a biological equivalent to *Cx. annulirostris* and *Cx. tritaeniorhynchus*, can travel 1250–1350 km [43]. Displacement of mosquitoes at night is approximately six-fold higher than during the day [27]. With the right environmental conditions, crepuscular-nocturnal mosquitoes take off after dusk and rapidly ascend in the upper air column to 100–300 m altitude [17, 27]. As *Cx. tritaeniorhynchus* is known to be most active 3 h after sunset, the species may be more prone to long dispersal distances at night because of its preferential feeding behaviour [32]. Given the propensity for a range of *Culex* species to travel lengthy distances between continental land masses, it remains plausible that direct entry into WA may have occurred from Timor-Leste. *Culex tritaeniorhynchus* mosquitoes detected in Doongan may represent a separate incursion from that reported in the NT, and possibly even Kununurra. As limited mosquito surveillance has been undertaken in this remote region, geographic distribution of the species may extend beyond the trapping sites described in this study. More extensive surveillance of the region, in addition to phylogenetic analysis of the Doongan specimens, is required to comment further on this hypothesized route of entry.

### Temporal abundance and geographic distribution in WA

*Culex tritaeniorhynchus* was detected at an increasing number of sites each year over the 38-month period. These results may reflect expansion of species distribution or simply that the trapping frequency increased. With the exception of the Doongan specimens, *Cx. tritaeniorhynchus* was observed in traps during the months of January to April in all years, coinciding with the peak wet season in Kununurra and the broader SWEK area [12]. It is expected that environmental conditions at this time of year (wet season) are more conducive to rainfall-driven mosquito breeding (Table 1). Trapping undertaken between November to December, and May to June, did not otherwise yield the species, coinciding with reduced rainfall [12]. Seasonal variation of the species has been well documented in the northern hemisphere. *Culex tritaeniorhynchus* populations have been noted to rise in late March and decrease in September/October across Korea, Japan and China, acknowledging northern and southern hemisphere seasonal differences [11, 34, 39]. Of note, the collection of specimens from Doongan was later than expected (May). The area reported unseasonably high rainfall the night trapping took place and a rapid rise in humidity over the 2 days leading up to the night of trapping, which may have contributed to the presence of the species at this unexpected time [12]. *Culex tritaeniorhynchus* is sensitive to photoperiod and temperature and known to survive adverse periods by reproductive diapause or quiescence [41, 50]. The latter generally involves a temporary suspension of normal activity initiated by, and continuing during, unfavourable conditions. The removal of these conditions allows a return to normal activity [9, 41]. Although it is not possible to reach a conclusion, the unseasonal downpour and sudden rise in humidity in the days leading up to the trapping event in Doongan could have rapidly brought *Cx. tritaeniorhynchus* out of their dry season quiescence.

### Public health implications

The Kimberley region of WA is considered high risk for a range of mosquito-borne viruses. Ross River virus (RRV) is the most frequently notified arboviral disease, followed by Barmah Forest virus (BFV) [33]. Although clinical infections are rare, flaviviruses Murray Valley encephalitis virus (MVEV) and West Nile virus Kunjn strain (WNV_KUN_) are also enzootic in the region [33]. Prior to 2000, arboviral encephalitis was associated with infections in indigenous populations, but as the mining and tourism industries expanded in northern WA, the risks to non-Aboriginal workers and tourists increased [44, 45]. This has been further exacerbated in recent years, following the first evidence of JEV activity in the region, coupled with detection of the highly competent JEV vector *Cx. tritaeniorhynchus* detailed in this study [8]. With a changing climate, arbovirus activity in WA is becoming more frequent and difficult to predict.

There remains a critical need to actively raise awareness of the public health risk associated with mosquitoes, particularly amongst cohorts of individuals identified as being at increased risk of acquiring a mosquito-borne disease in the Kimberley, including people living in Aboriginal communities and those undertaking outdoor occupational duties or recreational activities [37, 45]. Although *Cx. tritaeniorhynchus* preferentially feeds on cows and pigs, its anthropophagy increases when sleeping outdoors [55]. Individuals camping at high-risk times of the year should be encouraged to utilise bed netting and ensure their tents, caravans and accommodation have intact insect screening. People living in regional/remote Aboriginal communities should also be encouraged to use bed netting, where outdoor sleeping arrangements are in place [13]. Consideration also needs to be given to the important role that fishing has played in sociocultural practices by indigenous Australians for centuries, inadvertently bringing individuals in proximity to vector mosquito habitats [10, 48]. Given there are significant barriers to accessing timely healthcare services in remote regions, raising awareness and facilitating primary prevention practices (e.g. JEV vaccination) will play an important role in mitigating risk associated with flavivirus infections, improving health outcomes and promoting health equity in indigenous communities [20].

It is expected that *Cx. tritaeniorhynchus* is now established and will continue to thrive in the Kimberley region. The ongoing development of the Ord River Irrigation area in Kununurra provides habitat suitability to support the long-term establishment and year-round persistence of a range of *Culex* species, including *Cx. tritaeniorhynchus* [25]. The use of the land for extensive agriculture, coupled with the presence of artificially established irrigation channels, drainage ditches and tail water storage dams, has created semi-permanent and temporary ground pools with emergent vegetation. This environment is not dissimilar to irrigated rice fields, known to provide a primary larval habitat for *Cx. tritaeniorhynchus* in Southeast Asia [28, 46]. Surveillance officers regularly noted the presence of waterbirds, including ardeid species, along the Ord River Irrigation area over 2022–2023, often in very large numbers. Collectively, this environment provides an ideal site for vector mosquitoes and maintenance hosts of pathogenic flaviviruses, including JEV, to interact. Predicted suitability modelling supports this conclusion, indicating the Kimberley region is highly suitable for JEV transmission, based on the inclusion of wildlife reservoirs and vectors [24]. Due to the sheer extent of mosquito breeding habitat in the Kimberley region during the wet season, it is logistically too difficult to effectively manage larval populations using chemical control measures. However, management is more achievable in residential areas, such as those observed in this study around the Kununurra township, where *Cx. tritaeniorhynchus* is suspected of breeding and seeking harbourage in over-vegetated drainage infrastructure. Studies have demonstrated that the risk of mosquito-borne disease significantly increases for individuals living within 1–2 km of vector breeding habitats [26]. Constructed wetlands installed to improve urban drainage and storm water management can facilitate mosquito breeding and harbourage if not adequately designed or maintained [42]. A regular maintenance regime to clear the vegetation and encourage water to flow more freely may assist efforts to reduce the presence of *Cx. tritaeniorhynchus* and mitigate risk to public health in urban areas.

## Study limitations

Complimentary larval surveillance would have strengthened the study by confirming the location of *Cx. tritaeniorhynchus* breeding sites*.* Due to the rigorous mosquito trapping regime, insufficient resources were available to undertake this additional work at the time. All *Cx. tritaeniorhynchus* detections over the course of this study were in traps located within 550 m of predicted breeding habitats, which is consistent with current knowledge of species dispersal [38, 52, 53]. Future studies may wish to focus on adult mosquito trapping at incremental distances from known *Cx. tritaeniorhynchus* breeding sites to ascertain dispersal distances in WA. This information will help to inform risk to public health and mosquito management options.

Typically, accurate morphological identification should be undertaken before a leg is removed for molecular analysis to maintain the integrity of the specimen and ensure all identifying characters remain present. In this study, a leg was removed from each of the four specimens that underwent molecular analysis after preliminary morphological identification had been undertaken but prior to pinning for accurate identification. Although not ideal, it was necessary to confirm the identification of the four specimens by molecular analysis in this manner to progress with morphological identification of the remaining *Culex* specimens collected on 28 and 29 March 2022. Once molecular analysis had confirmed the species as *Cx. tritaeniorhynchus*, the remaining eight intact pinned specimens (collected in March 2022), were accurately identified under a Leica M80 dissecting microscope.

Finally, this study would have benefitted from undertaking molecular analysis of mosquitoes collected in Doongan, given these specimens were located the furthest distance from the original *Cx. tritaeniorhynchus* mosquitoes collected in Kununurra (March 2022) and were collected over 2 years later (May 2024). Unfortunately, as trapping in Doongan took place at the very end of the surveillance period, there was insufficient time and funding available to undertake further molecular analysis.

## Conclusions

The significance of *Cx. tritaeniorhynchus* establishment in the Kimberley region, namely SWEK, cannot be underestimated. Although *Cx. annulirostris* is ubiquitous across the region and is known to contribute to the transmission of pathogenic flaviviruses, it is highly possible that *Cx. tritaeniorhynchus* may have played a role in JEV transmission in both WA and the NT during the most recent outbreak in Australia. Although this study provides critical longitudinal data over a period of 38 months, ongoing surveillance is needed to provide a more comprehensive understanding of the true geographic distribution of *Cx. tritaeniorhynchus* in WA, temporal changes to distribution and abundance over the course of several mosquito seasons and the role this species may play in flavivirus transmission in the region.

## Supplementary Information

Additional file 1.

## Data Availability

No datasets were generated or analysed during the current study.
