# Correction: First detection of *Culex tritaeniorhynchus* in Western Australia using molecular diagnostics and morphological identification

**DOI:** 10.1186/s13071-024-06642-6

**Published:** 2025-01-16

**Authors:** Kimberly L. Evasco, Craig Brockway, Tamara Falkingham, Margaret Hall, Nerida G. Wilson, Abbey Potter

**Affiliations:** 1 Environmental Health Directorate, Western Australia Department of Health, 37 Kensington Street, East Perth, Perth, Western Australia 6004 Australia; 2 School of Biological Sciences, University of Western Australia, 35 Stirling Hwy, Crawley, Perth, Western Australia 6009 Australia; 3 Molecular Systematics Unit, WA Museum, 49 Kew St, Welshpool, Perth, Western Australia 6106 Australia

**Correction: Parasites & Vectors (2024) 17:500** 10.1186/s13071-024-06566-1

Following publication of the article [1], it was brought to the journal’s attention that due to typesetting errors, there were three errors in the article: in the Background section, reference 15 had been (incorrectly) written out in full rather than being cited as ‘[15]’; in the caption of Table 1, ‘traping set’ had been written in place of ‘trap set’; and in the subsection ‘Study limitations’, ‘550 of predicted breeding habitats’ had been written instead of ‘550 m of predicted breeding habitats’. The errors have since been corrected in the published article. The publisher thanks you for reading this erratum and apologizes for any inconvenience caused.
