# Supplementary material for: First detection of Culex tritaeniorhynchus in Western Australia using molecular diagnostics and morphological identification
Source: Parasit Vectors. 2024 Dec 4;17:500. doi: 10.1186/s13071-024-06566-1 (PMC11616137; doi:10.1186/s13071-024-06566-1)
Supplement: Supplementary file 1 — Additional file 1. [file 13071_2024_6566_MOESM1_ESM.pdf]

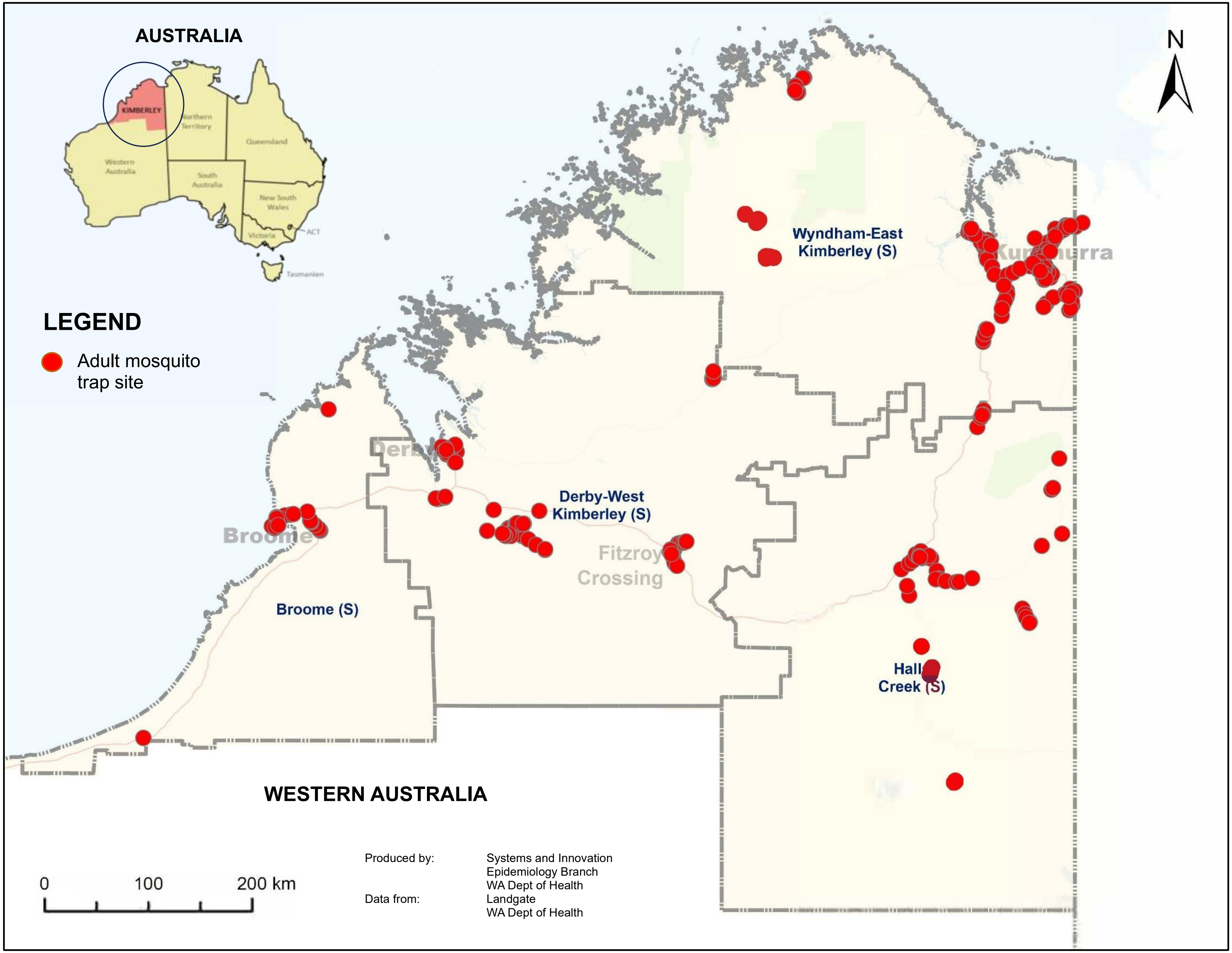
AUSTRALIA
KIMBERLEY
Northern Territory
Queensland
Western Australia
South Australia
New South Wales
Victoria
ACT
Tasmanien
LEGEND
Adult mosquito trap site
N
Wyndham-East Kimberley (S)
Kununurra
Derby
Derby-West Kimberley (S)
Broome
Fitzroy Crossing
Broome (S)
Halls Creek (S)
WESTERN AUSTRALIA
0
100
200 km
Produced by:
Systems and Innovation
Epidemiology Branch
WA Dept of Health
Data from:
Landgate
WA Dept of Health
